# Pancreaticopleural fistula causing recurrent bilateral pleural effusions: A case report

**DOI:** 10.1097/MD.0000000000041029

**Published:** 2024-12-20

**Authors:** Rixiang Liu, Ruolin Su, Haiyu Yan, Huangxin Zhu, Nuobei Zhang

**Affiliations:** a Department of Gastroenterology, The Second Affiliated Hospital of Nanchang University, Jiangxi, China.

**Keywords:** endoscopic treatment, ERCP, pancreatic pseudocyst, pancreaticopleural fistula, pleural effusion

## Abstract

**Rationale:**

Pancreaticopleural fistula (PPF) is an infrequent etiology of pleural effusion, characterized by nonspecific thoracic symptoms, which often leads to misdiagnosis and subsequent severe complications. Consequently, early diagnosis is crucial for effective management and the prevention of adverse outcomes. This report presents a rare case of PPF causing bilateral pleural effusions, aiming to enhance clinical recognition of this condition.

**Patient concerns:**

A 51-year-old male with a history of hepatitis B, pulmonary tuberculosis, and chronic pancreatitis presented with recurrent chest tightness, dyspnea, and significant weight loss. He had undergone multiple hospitalizations for pleural effusions with no definitive diagnosis.

**Diagnoses:**

The patient exhibited a marked increase in amylase levels within the pleural effusion, and magnetic resonance cholangiopancreatography revealed a pancreatic pseudocyst herniating into the mediastinum. Endoscopic retrograde cholangiopancreatography (ERCP) demonstrated mild dilation of the pancreatic duct, leading to a definitive diagnosis of PPF.

**Interventions:**

Despite initial conservative measures, including thoracentesis, antimicrobial therapy, and somatostatin analogs, the patient continued to experience persistent pleural effusion. Ultimately, ERCP with pancreatic duct stent placement was performed, leading to a significant improvement in the patient’s condition.

**Outcomes:**

Re-evaluation 2 months postdischarge using a thoracoabdominal computed tomography scan confirmed near-complete resolution of the pancreatic pseudocyst, absence of pleural effusion, and normalization of pancreatic function.

**Lessons:**

This case underscores the importance of a multidisciplinary approach in diagnosing and managing PPF. It highlights the utility of ERCP in both diagnosing and treating PPF and the need for early recognition to prevent diagnostic delays and improve patient outcomes.

## 1. Introduction

Pancreaticopleural fistula (PPF) is a rare cause of pleural effusion, distinct from the self-limited, small amounts of effusion caused by acute pancreatitis. PPF-induced pleural effusion is typically large and recurrent, predominantly occurring in a minority of patients with chronic pancreatitis.^[1]^ Patients often exhibit nonspecific symptoms, such as chest pain, dyspnea, cough, or fever,^[2,3]^ which are similar to other thoracic diseases, while signs and symptoms related to pancreatitis are often inconspicuous, making misdiagnosis and delayed diagnosis highly likely. This study retrospectively analyzes the clinical data of a patient with PPF causing recurrent bilateral pleural effusions and reviews the relevant literature to provide experience for the diagnosis and treatment of similar cases.

## 2. Case report

The 51-year-old male patient was admitted to the hospital with a 5-month history of recurrent chest tightness and pain, which had acutely worsened with dyspnea over the preceding day. His medical history comprised hepatitis B, pulmonary tuberculosis, and recurrent episodes of acute pancreatitis since 2015, along with a pancreatic pseudocyst. He had a 20-year smoking history, but there was no record of alcohol abuse. Five months prior, the patient experienced unexplained chest tightness and pain, occasional abdominal distension, poor appetite, and significant weight loss, without chills, fever, nausea, vomiting, abdominal pain, or melena. At an outside hospital, a chest-color Doppler ultrasound revealed a large amount of pleural effusion on the left side. Thoracentesis drained a large amount of bloody fluid, and the patient was discharged after symptomatic treatment with pleural drainage, anti-infection, acid suppression, and somatostatin analogs. One month before admission to our hospital, the patient had bilateral pleural effusions and was treated with thoracentesis, which showed bloody exudative fluid, with lactate dehydrogenase 989.10 U/L and amylase 7279.80 U/L. The t-SPOT test was positive, and the tuberculosis antibody was negative. Considering the patient’s history of pulmonary tuberculosis and significant weight loss, tuberculous pleurisy was suspected, and tumorous pleurisy could not be ruled out. The patient was advised to undergo a thoracoscopy, biopsy, and PET-CT for further clarification, but he refused further diagnosis and treatment and was discharged after diagnostic anti-tuberculosis treatment (Isoniazid 0.3 g qd, Rifampicin 0.45 g qd, Pyrazinamide 0.5 g tid, Ethambutol 0.75 g qd). One day before admission, the patient’s chest tightness and pain recurred and gradually worsened, accompanied by dyspnea, and he was admitted to the Respiratory Department of our hospital. Physical examination: stable vital signs, clear consciousness, weakened fremitus in the left chest, and dull sound on lower percussion. Left lung breath sounds are weak, right lung breath sounds are coarse, and neither lung has obvious dry or moist rales, no significant abnormalities in cardiac and abdominal examination, and no edema in the lower limbs.

**Figure 1.**
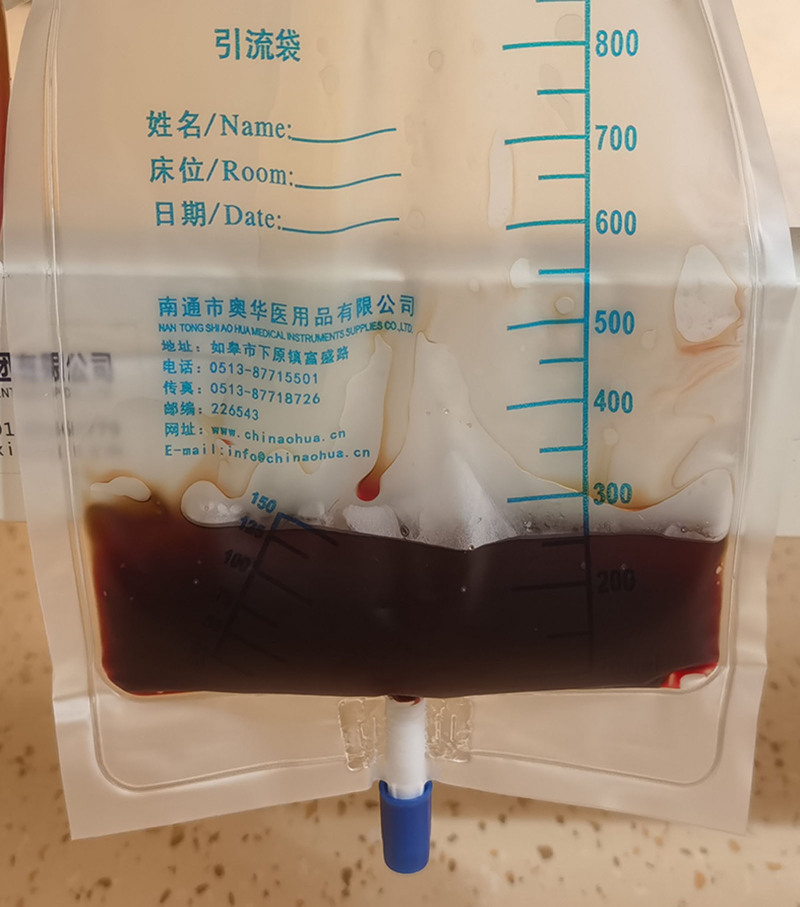
Hemorrhagic pleural effusion.

Post-admission pancreatic function tests: serum amylase 759.80 U/L, lipase 965.5 U/L, pancreatic amylase 662.30 U/L; CRP: 13.4 mg/L; routine blood tests, biochemistry, cardiac infarction triad, coagulation function, etc, were roughly normal. Chest water color Doppler ultrasound: in the left thoracic cavity, about 110 mm of free fluid dark area was detected; in the right thoracic cavity, about 27 mm of free fluid dark area was detected. Thoracic closed drainage was immediately performed, and bloody pleural effusion was drained (Fig. 1). Pleural fluid routine: turbid, no clots, reddish-brown, Lee–White test positive 2+, red blood cells 22,000 × 10^6^/L, white blood cells 748 × 10^6^/L, polymorphonuclear neutrophils 80%, mononuclear cells 15%, pleural fluid biochemistry: total protein 55.70 g/L, lactate dehydrogenase 546.10 U/L, adenosine deaminase 28.10 U/L, amylase: 18,362.00 U/L, pleural fluid tumor quadruple test and carcinoembryonic antigen determination: no abnormalities; pleural fluid cytology: neutrophils, lymphocytes, mesothelial cells, and tissue cells were visible under the microscope, no malignant tumor cells or abnormal and mitotic figures were seen.

**Figure 2.**
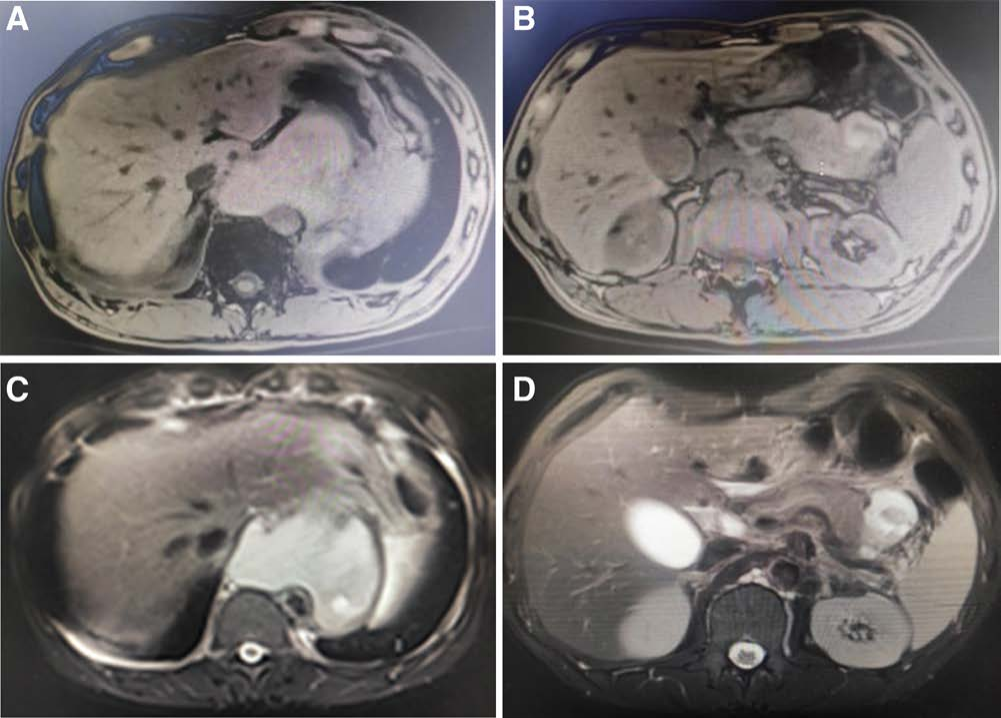
Abdominal MRI (following thoracentesis and drainage): encapsulated fluid in the pancreatic tail-posterior mediastinal area, which is indicative of a pancreatic pseudocyst herniating into the mediastinum. (A, B) T1-weighted image; (C, D) T2-weighted image. MRI = magnetic resonance imaging.

**Figure 3.**
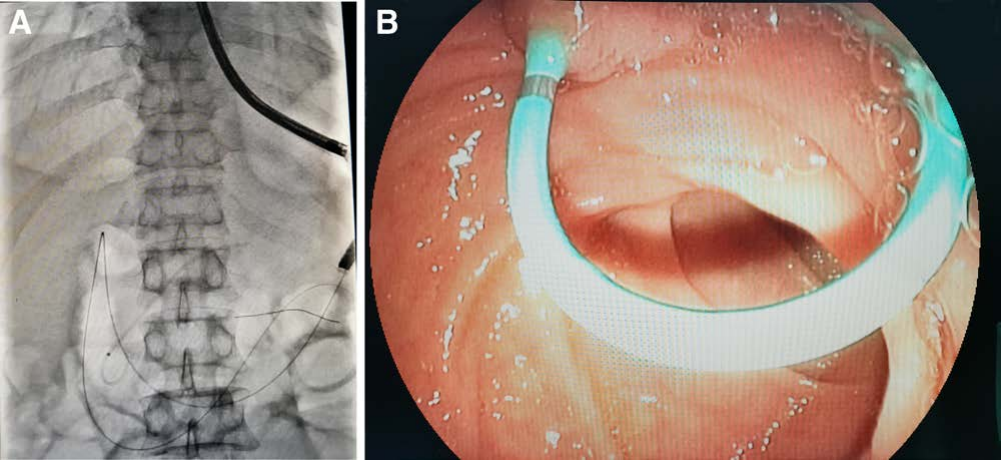
(A) Endoscopic retrograde cholangiopancreatography (ERCP) via the papilla of Vater demonstrates mild dilation of the pancreatic duct without significant contrast leakage. (B) An 8 cm × 5F pancreatic duct stent is placed. ERCP = endoscopic retrograde cholangiopancreatography.

**Figure 4.**
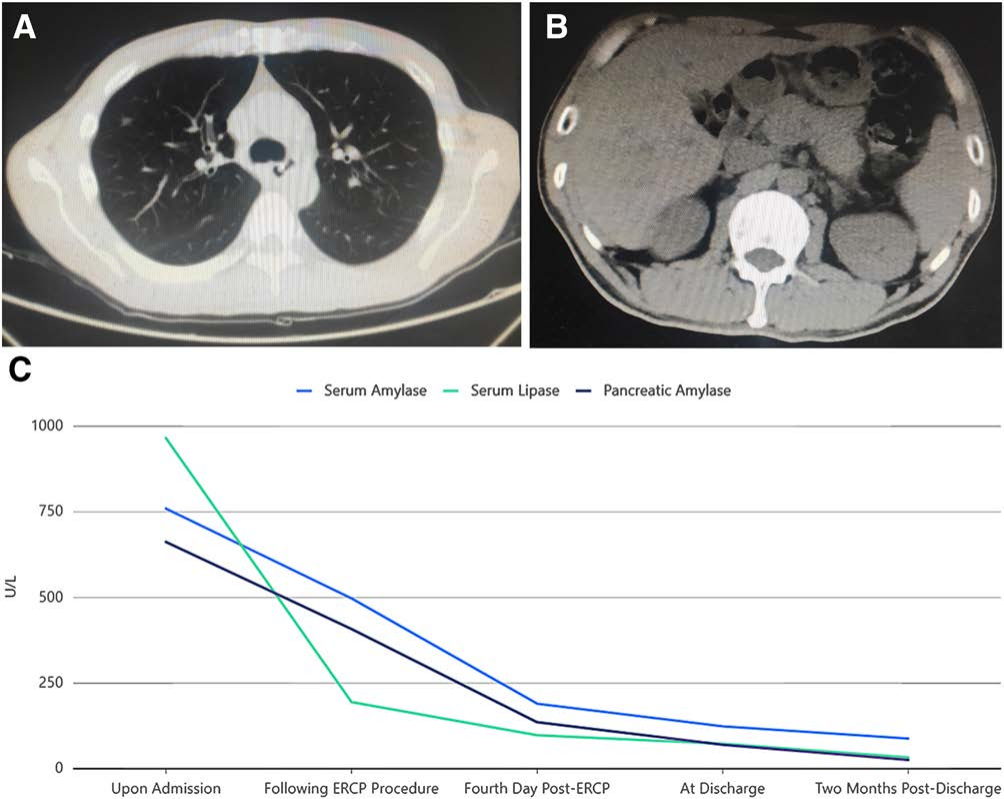
(A) Follow-up chest CT 2 months after discharge shows near-complete absorption of the original bilateral pleural effusions. (B) Abdominal CT demonstrates near-complete absorption of the pseudocyst in the pancreatic tail-posterior mediastinal area and peripancreatic exudate. (C) The patient’s pancreatic function was gradually returning to normal. CT = computed tomography, ERCP = endoscopic retrograde cholangiopancreatography.

Post-thoracentesis, thoracoabdominal MRI + MRCP showed encapsulated fluid in the pancreatic tail-posterior mediastinum area, suggesting the herniation of pancreatic pseudocyst into the mediastinum with no dilated pancreatic duct (Fig. 2). After comprehensive consultation with the Department of Gastroenterology, Hepatobiliary Surgery, and Imaging, the possibility of pancreatic pleural fistula was considered, and the patient was transferred to the Department of Gastroenterology for endoscopic retrograde cholangiopancreatography (ERCP; Fig. 3). The endoscope showed a papillary duodenal papilla with a granular opening. Biliary cannulation showed mild dilation of the pancreatic duct, with no obvious leakage of contrast medium. An 8CM × 5F pancreatic duct stent was placed, and pancreatic juice drainage was unobstructed. A nasogastric jejunal tube was placed through the nose. The patient did not complain of any special discomfort after the operation and was treated with fasting, parenteral nutrition, octreotide, and broad-spectrum antibiotics, with dynamic monitoring of blood amylase. After the disappearance of pleural drainage fluid, the drainage tube was removed, and the patient was discharged. Two months after discharge, the patient returned for reexamination, and the thoracoabdominal computerized tomography (CT) showed that the pancreatic pseudocyst and peripancreatic exudate were absorbed, with no pleural effusion, and pancreatic function returned to normal (Fig. 4).

## 3. Discussion

Pancreaticopleural fistula is a rare complication of acute or chronic pancreatitis caused by pancreatic inflammation or traumatic injury. Most case reports have proven to be related to chronic pancreatitis induced by alcohol, and other rare causes such as gallstones, trauma, iatrogenic injury, autoimmune pancreatitis, and pancreatic anatomical abnormalities are also reported from time to time.^[4–6]^ The incidence of PPF in patients with chronic pancreatitis is 0.4%, and it can reach 4.5% in patients with pancreatic pseudocysts.^[7–9]^

The formation mechanism of PPF is mostly related to the rupture of the main pancreatic duct. The rupture of the duct on the front surface of the pancreas usually causes ascites, while the rupture of the duct on the back surface of the pancreas leads to pleural effusion. The rupture of the back surface of the pancreatic duct allows pancreatic secretions and necrotic materials to have the opportunity to transfer to the mediastinum through the aortic and esophageal hiatus with smaller pressure, forming a mediastinal pseudocyst. When the mediastinum or diaphragm has a tiny defect to form a fistula, it will lead to pleural effusion.^[9,10]^ Due to the anatomical position of the pancreas, pleural effusion caused by PPF is mostly in the left pleural cavity, and a few involve the right pleural cavity or bilateral pleural cavities.^[6,8]^ This article shares a case of bilateral pleural effusion.

PPF-induced pleural effusion is usually large in volume and recurrent. Many patients present with symptoms such as dyspnea, cough, and chest pain to the hospital.^[1–3]^ To alleviate the patient’s symptoms and clarify the cause of pleural effusion, thoracentesis has become an essential diagnostic and therapeutic means. Patients with PPF usually drain a large amount of exudative fluid rich in amylase, which is the key to diagnosing PPF. An average amylase level of 18,450 IU/L was reported in a study report (range: 1830–164,187 IU/L).^[11]^ At present, there is no clear diagnostic threshold value for pleural effusion amylase in PPF, but when the level exceeds 1000 U/L, excluding diseases such as acute pancreatitis, tuberculosis, tumors, esophageal rupture, liver cirrhosis, and hydronephrosis that can also cause increased amylase in pleural effusion, the pleural effusion should be suspected to be related to the pancreas.^[10]^ Especially when draining pleural effusion rich in amylase, PPF should be actively considered.^[12]^

CT scan is often used as a routine imaging examination for diagnosing thoracoabdominal diseases because of its short examination time, wide popularity, and high patient acceptance. It can observe atrophy and calcification of the pancreatic parenchyma, pseudocysts, and ductal dilation.^[8]^ According to the latest research report, 63% of PPF patients were found to have fistulae after CT examination.^[6]^

Magnetic resonance cholangiopancreatography (MRCP) is often used as the preferred imaging examination for diagnosing PPF due to its noninvasive nature and high fistula detection rate (83%).^[6]^ It can not only detect fistulae under severe pancreatic duct stenosis but also draw the anatomical map of the pancreatic duct and observe changes in nearby structures, providing an important basis for formulating the best treatment plan for patients.^[4]^

ERCP, as an invasive examination, has potential complications such as bleeding, perforation, and acute pancreatitis, and its success rate is affected by the operator’s technical level and the complexity of the pancreatic anatomy. It is usually not the first diagnostic tool. However, ERCP can observe the pancreatic papilla and surrounding anatomical structures under direct endoscopic vision and can treat interventions while detecting fistulae, so it is recommended to consider ERCP when the MRCP examination fails.^[13]^

It can be seen that diagnosing PPF is not easy. For this special patient, the trajectory of the disease is particularly complex and enlightening: Since 2015, the patient has had a history of recurrent pancreatitis, and the first diagnosis also had the formation of pancreatic pseudocysts, indicating that the patient has a long-term chronic pancreatic damage; The patient has been hospitalized several times for pleural effusion, and the drainage of pleural effusion is bloody, with a significant increase in amylase content in the chest water, while tumor-related indicators are not abnormal, and thoracic imaging examination does not see obvious occupation, which can rule out chest water abnormalities caused by malignant tumors; The patient has a history of pulmonary tuberculosis for many years, and the admission T-SPOT test is positive. Although this background increases the complexity of the diagnosis, thoracic CT scanning does not see recurrent lesions, sputum smears and culture do not see mycobacteria, and adenosine deaminase is normal, making the basis for the diagnosis of tuberculous pleurisy weak. One month of diagnostic anti-tuberculosis treatment is not effective, and the recurrence of pleural effusion further weakens the assumption of tuberculous pleurisy; Imaging examination can see mediastinal pseudocysts; although the existence of fistulae is not directly observed, it provides new clues for the source of pleural effusion. Combining the patient’s clinical manifestations, chest water characteristics, and evaluation of treatment effects, we highly suspect that the pleural effusion is caused by the leakage of mediastinal pseudocysts, thus preliminary establishing the direction of PPF diagnosis. After several conservative treatments failed to achieve significant effects, we decisively took endoscopic pancreatic duct stent implantation to drain pancreatic juice, and the therapeutic effect was obvious, thus confirming the diagnosis of PPF.

The treatment methods for PPF include conservative drug treatment, endoscopic treatment, and surgical treatment. The specific plan should be based on the patient’s situation. Conservative treatment includes fasting, total parenteral nutrition, anti-infection, inhibition of pancreatic enzyme secretion, and thoracentesis to inhibit pancreatic activity, reduce pancreatic juice secretion, promote the absorption of pleural effusion, and thus close the fistula of pseudocysts and pancreatic ducts. However, the prevailing literature indicates that the success rate of conservative treatment is generally low.^[11,14,15]^ Wronski et al^[9]^ suggested that conservative treatment should be considered when the patient’s main pancreatic duct is dilated but not ruptured or narrowed. Most previous articles suggest considering ERCP after 2 to 3 weeks of conservative treatment,^[4,11]^ and some suggest that ERCP is needed after 1 week of ineffective conservative treatment.^[15]^ In the case we reported, although the patient’s ERCP only showed mild dilation of the pancreatic duct, the patient still had recurrent pleural effusion after several hospitalizations for conservative treatment. Therefore, we suggest that once the diagnosis of PPF is clear, endoscopic treatment can be performed after excluding contraindications. We suggest conservative drug treatment as an adjuvant treatment for endoscopic and surgical treatment. Considering the substantial time elapsed from the patient’s admission to the establishment of a definitive diagnosis and given that conservative treatment has been concurrently implemented during this period, persisting with conservative measures without noticeable therapeutic benefits could potentially prolong the patient’s hospital stay and heighten the risk of complications.

The success rate of endoscopic treatment for PPF is usually more than 90%, especially ERCP with transpapillary drainage and pancreatic duct stent placement, which has become the preferred treatment option for PPF.^[3]^ The main purpose of pancreatic duct stent placement is to block and avoid the abnormal connection between the pancreatic duct and the pleura, and to provide a path to ensure that pancreatic secretions can flow into the duodenum.^[15]^ If the main pancreatic duct has severe stenosis or rupture and it is difficult to place a stent through the papilla, it is possible to consider an endoscopic ultrasonography-guided percutaneous (through the upper gastrointestinal wall) approach.^[3]^ In the latest report, Kitagawa et al^[16]^ successfully used the “Tunneling technique” to place an nasopancreatic drainage tube in a place with severe downstream pancreatic duct stenosis, thus successfully treating the fistula, which provides a new idea for the endoscopic treatment of PPF.

For patients who are ineffective for drug and endoscopic treatment, surgical treatment may be needed, such as distal pancreatectomy and pancreatojejunostomy. With the development of endoscopic technology, surgical treatment is rarely used for PPF treatment and is usually considered as the last treatment option. However, in some cases, early surgical intervention may be a more appropriate choice. For endoscopic treatment failure or high-risk cases, surgical intervention should not be delayed to avoid increasing the incidence and mortality of patients.^[9]^

Therefore, in clinical practice, the treatment plan should be adjusted according to the patient’s specific situation and disease progression to achieve the best treatment effect.

## 4. Conclusion

In summary, PPF patients often have no specific performance, mostly chest and lung disease symptoms, and pancreatitis symptoms and signs are rare. Therefore, to avoid diagnostic delays, when patients have recurrent large amounts of pleural effusion, they should first be asked in detail whether they have a history of chronic pancreatitis or long-term alcohol consumption, and then perform thoracentesis. When pleural effusion with a high concentration of amylase is drained, after excluding the possibility of other diseases such as acute pancreatitis, tumors, and tuberculosis, PPF should be actively considered. Subsequently, further imaging examinations such as CT and MRCP should be improved, and if fistulae and pseudocyst herniation into the mediastinum are shown, the diagnosis of PPF can be confirmed. If there is doubt in the diagnosis of a single discipline, it is recommended to have a multidisciplinary consultation to further clarify the diagnosis. The treatment of PPF should be based on the patient’s situation to establish an individualized plan, and it is recommended to first choose endoscopy combined with drug treatment. If endoscopic treatment fails, surgical treatment should be considered as soon as possible.

## Author contributions

**Conceptualization:** Rixiang Liu, Ruolin Su.

**Data curation:** Haiyu Yan.

**Investigation:** Rixiang Liu, Ruolin Su, Haiyu Yan, Huangxin Zhu.

**Writing – original draft:** Rixiang Liu.

**Writing – review & editing:** Nuobei Zhang.
